# Molecular and Biological Characterization of a Newly Identified Virus Representing a Novel Taxon of *Alphaflexiviridae* Infecting Different Accessions of Seashore Paspalum, a Turfgrass, Widely Grown in the United States

**DOI:** 10.3390/ijms27135760

**Published:** 2026-06-26

**Authors:** Sayanta Bera, Taylor F. Schulden, Xiaojun Hu, Peter Abrahamian, Yu Yang, Anna L. Paulson, Amy Harvey-White, Shreena Pradhan, Katrien Devos, Christina Devorshak, Joseph A. Foster, Bishwo N. Adhikari

**Affiliations:** 1 United States Department of Agriculture (USDA), Animal and Plant Health Inspection Service (APHIS), Plant Protection and Quarantine (PPQ), Plant Germplasm Quarantine Program (PGQP), Beltsville, MD 20708, USA; taylor.schulden@usda.gov (T.F.S.); xiaojun.hu@usda.gov (X.H.); yu.yang@usda.gov (Y.Y.); annapaulson21@gmail.com (A.L.P.); amy.harvey-white@usda.gov (A.H.-W.); joseph.a.foster@usda.gov (J.A.F.); 2 USDA-ARS National Germplasm Resources Laboratory, Beltsville, MD 20705, USA; peter.abrahamian@usda.gov; 3 Institute of Plant Breeding, Genetics and Genomics, Athens, GA 30602, USA; shreena.pradhan@uga.edu (S.P.); kdevos@uga.edu (K.D.); 4 Department of Crop and Soil Sciences, University of Georgia, Athens, GA 30602, USA; 5 Department of Plant Biology, University of Georgia, Athens, GA 30602, USA; 6 USDA-APHIS-PPQ, Field Operations, Raleigh, NC 27606, USA; christina.devorshak@usda.gov

**Keywords:** *Alphaflexiviridae*, *Paspalovirus*, *Paspalum vaginatum*, viral genomics, latent infection, turfgrass, molecular diagnostics, virus evolution

## Abstract

Seashore paspalum (*Paspalum vaginatum*), a salinity-tolerant turfgrass, lacks well-characterized viral profiles. This study reports the discovery of a novel virus, tentatively named Paspalum latent virus (PaLV), representing a new taxon within the *Alphaflexiviridae*. Using high-throughput sequencing and RACE PCR, the 6995 nt genome was determined, revealing five open reading frames. Notably, PaLV lacks the AlkB domain and exhibits unique features, including overlapping start-stop codons (ORF4/ORF5) and a second in-frame AUG in the coat protein (CP) region. Phylogenetic analysis of the replicase placed PaLV in a distinct clade, separate from *Potexvirus* and *Lolavirus*. Despite low sequence identity, AlphaFold2 revealed conserved CP structural domains. Genetic analysis of 11 isolates showed low diversity and strong purifying selection. Pathogenicity assays through mechanical transmission demonstrated a broad but latent host range, including *Zea mays* and *Sorghum* spp. These findings suggest PaLV represents a novel species within a putatively new genus, *Paspalovirus*. Given its 90% incidence rate and latent profile, the RT-PCR assay developed here is vital for routine molecular diagnostics in turfgrass management and germplasm conservation.

## 1. Introduction

Turfgrasses play a vital role in environmental protection and human well-being [1]. Seashore paspalum (*Paspalum vaginatum* Sw.), a warm-season perennial turfgrass, has attracted attention due to its salinity tolerance and adaptability to coastal environments in tropical and subtropical regions [2,3]. As a result, seashore paspalum is a popular choice for sports fields in coastal regions where freshwater availability is an issue and saltwater irrigation results in tremendous cost savings [4]. Beyond its functional benefits, seashore paspalum also contributes to environmental sustainability by mitigating the urban heat island effect, providing forage and aesthetic value, and generating economic and social benefits. The species is also reportedly tolerant to flooding and low-oxygen conditions [1,5,6]. Due to these numerous benefits, the cultivation area of *Paspalum* has expanded beyond coastal areas; however, in new environments, plants are exposed to many unknown pathogens, increasing the risk of disease damage.

Seashore paspalum, similar to other turfgrasses, is propagated through clonal methods, resulting in genetic uniformity [7]. As a result, viruses are easily spread across the propagated plant material and introduced into new areas [7,8]. Notable viruses that have been identified in *Paspalum*, under natural or experimental conditions, include Paspalum striate mosaic virus (PSMV, *Geminiviridae*) [9,10], sugarcane mosaic virus (SCMV, *Potyviridae*) [11], sugarcane streak Réunion virus (SSRV, *Geminiviridae*) [12], Chloris striate mosaic virus (CSMV, *Geminiviridae*) [9], Paspalum dilatatum striate mosaic virus (PDSMV, *Geminiviridae*) [9], and barley yellow dwarf virus (BYDV, *Tombusviridae*) [13].

In recent years, high-throughput sequencing (HTS) has emerged as a transformative technology in the field of virology, particularly in the realm of viral discovery and diagnostics [14,15]. This approach has led to the identification of numerous novel viruses in diverse biological samples, including grasses, weeds, trees, and food crops [16,17,18,19,20]. Therefore, in this study, we leveraged HTS to investigate the virome of *Paspalum* germplasm used in breeding programs. For this purpose, 27 *P. vaginatum* and three *P. distichum* (a sister species of *P. vaginatum*) accessions were obtained from the USDA National Plant Germplasm System (NPGS) collection and maintained at the University of Georgia (UGA), Griffin and Athens campuses.

Our results include the first report of a virus representing a novel taxon within the *Alphaflexiviridae* that infects both *P. vaginatum* and *P. distichum*. Confirmation of putative viral sequences was obtained by RT-PCR, and genome terminals were completed with 5′ RACE. In this study, we characterize this virus using phylogenetic, genomic structure, and biological analyses. Based on phylogenetic analysis and genomic structure, here we report a novel virus, tentatively named *Paspalovirus paspali* (Paspalum latent virus, PaLV), in the family *Alphaflexiviridae*.

## 2. Results

### 2.1. Genome Structure of PaLV

High-throughput sequencing and 5′ RACE revealed the complete genome of PaLV to be 6995 nucleotides (nt) in length, excluding the poly(A) tail. The 3′ terminus was confirmed to be complete as evidenced by the presence of a poly(A) tail sequence in the assembled contig, indicating the natural end of the viral genome. The genome comprised five open reading frames (ORFs), flanked by an 81-nt 5′ untranslated region (UTR) and an 86-nt 3′ UTR. Pairwise comparisons of PaLV ORFs with representative *Alphaflexiviridae* viruses showed the highest identity with Lolium latent virus (LoLV), ranging from 49 to 57% at the nucleotide level and 26–55% at the amino acid level (Table 1).

**Figure 1:**
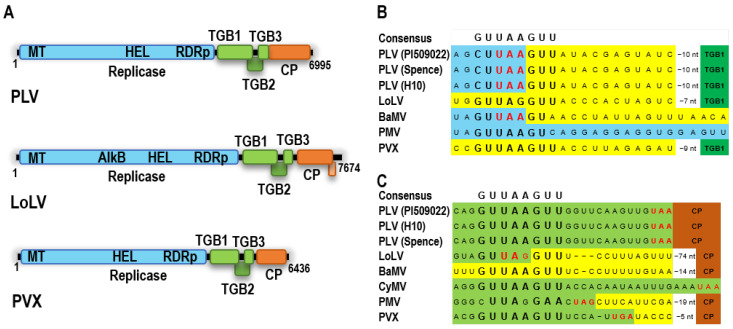
Diagram showing the genome organization of PaLV, LoLV, and PVX (**A**) and putative promoter sequences for sgRNAs of several viruses from the genera, in Lolavirus and Potexvirus (**B**,**C**). (**A**) The five proteins encoded by the viral genome are indicated, and in LoLV, the extra small overlapping ORF with CP is also shown. (**B**,**C**) Alignment of the putative promoter sequences upstream of TGB1 (**B**) and CP (**C**). The consensus sequences at the top, as reported in [23], are indicated. Light blue and deep green colors denote RdRp and TGB1 encoding regions, respectively (**B**). Light green and brown colors denote TGB3 and CP encoding regions, respectively (**C**). The yellow color indicates the intergenic regions, and the three red nucleotides indicate the stop codons. BaMV: Bamboo mosaic virus (NC_001642); CyMV: Cymbidium mosaic virus (NC_001812); LoLV: Lolium latent virus (EU489641); PMV: Papaya mosaic virus (NC_001748); PaLV: Paspalum latent virus; PVX: Potato virus X (MT752888).

ORF1 encoded a 173 kDa replicase protein containing conserved domains: Methyltransferase (aa 39–329), Viral RNA helicase (aa 768–997), and RNA-dependent RNA polymerase (aa 1178–1474) (Figure 1A). The AlkB domain, which is present in LoLV, was absent in ORF 1 of PaLV. ORFs 2–4 encoded triple gene block (TGB) proteins: TGB1 (30.4 kDa), TGB2 (13.5 kDa), and TGB3 (7.8 kDa). These proteins have been shown to play a crucial role in virus movement [21]. A putative octanucleotide promoter sequence for sgRNA1 (CUUAAGUU) was identified 21 nt upstream of ORF2, overlapping the stop codon of ORF1 (Figure 1B).

ORF5 encoded the coat protein (CP) of 35.4 kDa with a start codon in-frame and immediately followed the stop codon of ORF4, with no intergenic region (Figure 1C). A putative sgRNA2 promoter sequence (GUUAAGUU) was located 14 nt upstream of ORF5, within ORF4. ORF6, present in LoLV and other tentative lolaviruses such as Weeping alkaligrass lolavirus 1 (WaLoV1), that overlaps the 3′ end of ORF 5, was not identified in PaLV or the other tentative lola-like members, such as Foxtail millet lolavirus 1 (FmLoV1) (Figure 1A) [22].

### 2.2. Comparison of CP_PaLV_ with CP_LoLV_

**Figure 2:**
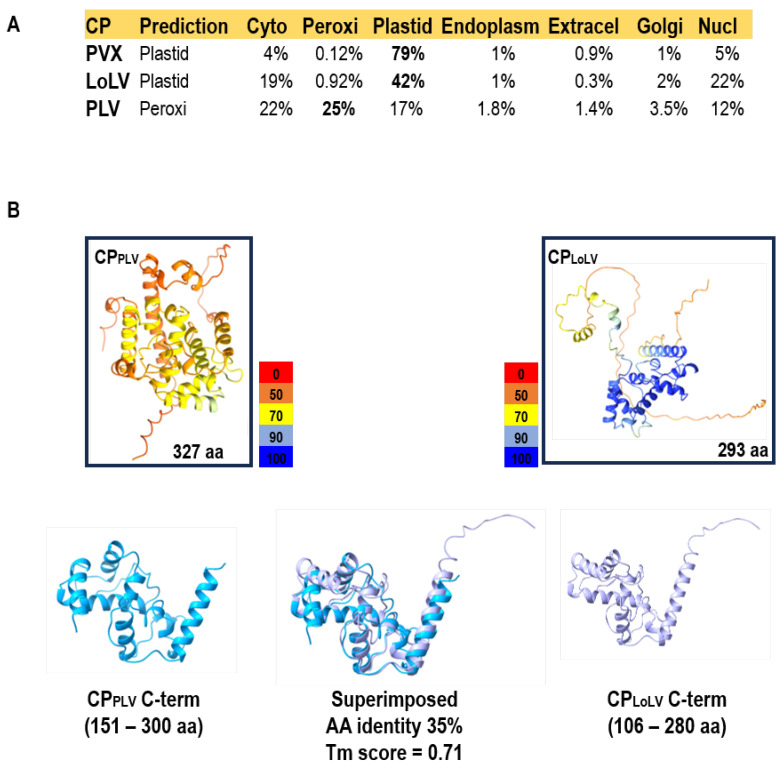
Comparison of CP_PaLV_ with CP_LoLV_. (**A**) A table showing the predicted localization of CP_PVX_, CP_PaLV_, and CP_LoLV_ in a plant cell as determined on a web server, Plant-mSubP. Percentages indicate the predicted probability of localization to each of the seven listed compartments; for each CP, the bolded value indicates the organelle with the highest predicted localization probability (i.e., the predicted destination). (**B**) Structure modeling of CP using Alphafold 2. Full-length structures of CP_PaLV_ and CP_LoLV_ are boxed. The numerical value shown in the bottom right corner indicates the number of aa in the CP. Color coding from red to blue indicates low to high structural confidence, respectively. The core and C-terminal regions of the CP_PaLV_ are superimposed on the corresponding CP_LoLV_ region with a high TM score.

Subcellular localization prediction using Plant-mSubP indicated that CP_PaLV_ was likely localized in the cytoplasm or peroxisomes, while CP_LoLV_ and CP_PVX_ were predicted to localize in plastids. Subsequently, the protein structures of CP_PaLV_ and CP_LoLV_ were modeled to compare their structural relatedness. For this purpose, both amino acid sequences were subjected to Alphafold2 protein structure predictions (Figure 2). The structure of CP_PaLV_ was predicted with a lower confidence level than that of CP_LoLV_ (Figure 2). The structural modeling of both CPs revealed the presence of three domains: (i) flexible N-terminal, (ii) the core, and (iii) C-terminal extensions. The N-terminus of PaLV does not contain a proteolytic cleavage site for the chloroplast transit peptide that yields a smaller CP isoform in LoLV. However, a smaller ORF of 28 kDa was predicted within the CP ORF. The core and C-terminal regions of the CP play crucial roles in protein-protein and protein-RNA interactions and have been found to show the highest degree of structural conservation in *Alphaflexiviridae* [24]. Consequently, a comparative analysis of the core and C-terminal domains (151–300 and 106–280 aa in CP_PaLV_ and CP_LoLV_, respectively) of both CPs was conducted. As predicted, a high TM score, indicative of high structural similarity, was obtained upon the superimposition of the core and C-terminal regions of both CPs, which consist of conserved α-helical secondary structures (Figure 2).

### 2.3. Phylogenetic Analysis

Maximum-likelihood phylogenetic analysis based on replicase amino acid sequences placed PaLV in a distinct clade, clearly separated from the *Potexvirus* and *Lolavirus* genera. The analysis also included putative viral sequences obtained through data mining and submitted as Third-Party Annotation (TPA), such as Saltwater paspalum lolavirus 1 (SpLoV1, synonym PaLV [22], FmLoV1, a Lola-like virus from Maize (Lola maydis), and WaLoV1. Their inclusion further supports the formation of this novel group within the *Alphaflexiviridae* family.

**Figure 3:**
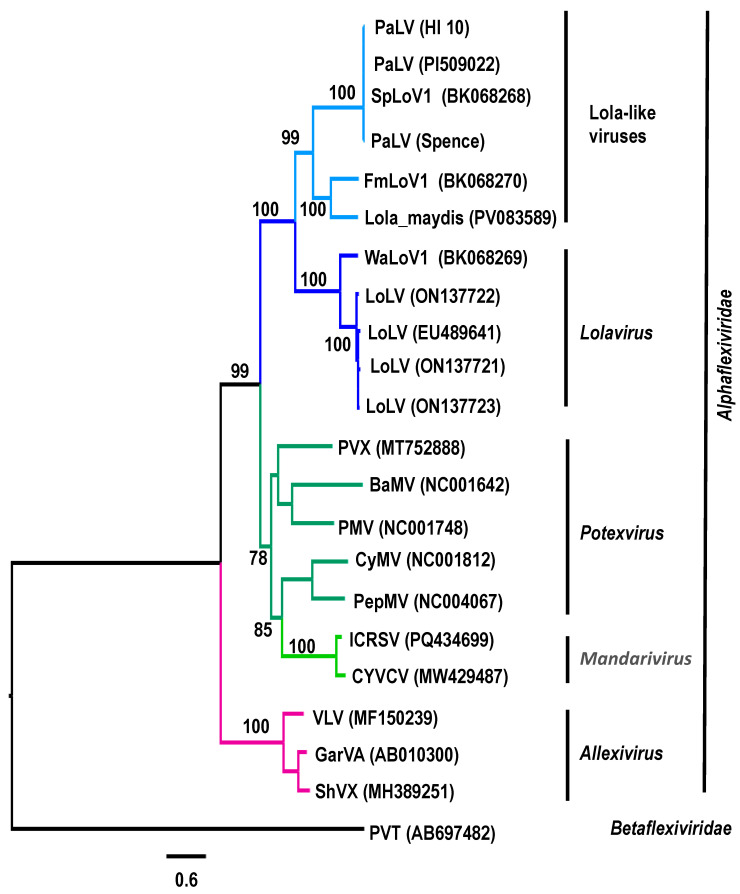
Maximum-likelihood phylogenetic tree based on aa sequences of replicase of plant-infecting viruses from the *Alphaflexiviridae* family. The branch number indicates bootstrap support in percentage (out of 1000 replicates). Branch colors denote distinct genera within the *Alphaflexiviridae* family: light blue, Lola-like viruses; dark blue, *Lolavirus*; green, *Potexvirus*; teal, *Mandarivirus*; magenta, *Allexivirus*. The scale bar at the bottom denotes amino acid substitutions per site. The tree is rooted to an outgroup, Potato virus T (PVT), from the *Betaflexiviridae* family. Abbreviations: BaMV—Bamboo mosaic virus; CYVCV—Citrus yellow vein clearing virus; CyMV—Cymbidium mosaic virus; FmLoV1—Foxtail millet lolavirus 1; GarVA—Garlic virus A; ICRSV—Indian citrus ringspot virus; LoLV—Lolium latent virus; Lola_maydis—Lola-like virus from maize retrieved from third party annotation database; PaLV: Paspalum latent virus; PepMV—Pepino mosaic virus; PMV—Papaya mosaic virus; PVX—Potato virus X; ShVX—Shallot virus X; SpLoV1— Saltwater paspalum lolavirus 1; VLV—Vanilla latent virus; WaLoV1—Weeping alkaligrass lolavirus 1.

All PaLV isolates along with SpLoV1formed a unique and well-supported clade (Figure 3). Notably, SpLoV1 shared 97% nucleotide identity with PaLV, which indicates the same viral species. FmLoV1 and Lola maydis sequences represent two potential new members within the PaLV clade. Whereas WaLoV1 appeared to cluster within the *Lolavirus* clade. No recombination events were detected in PaLV genomes using any of the algorithms implemented in RDP5.

### 2.4. Genetic Diversity of PaLV Population

**Figure 4:**
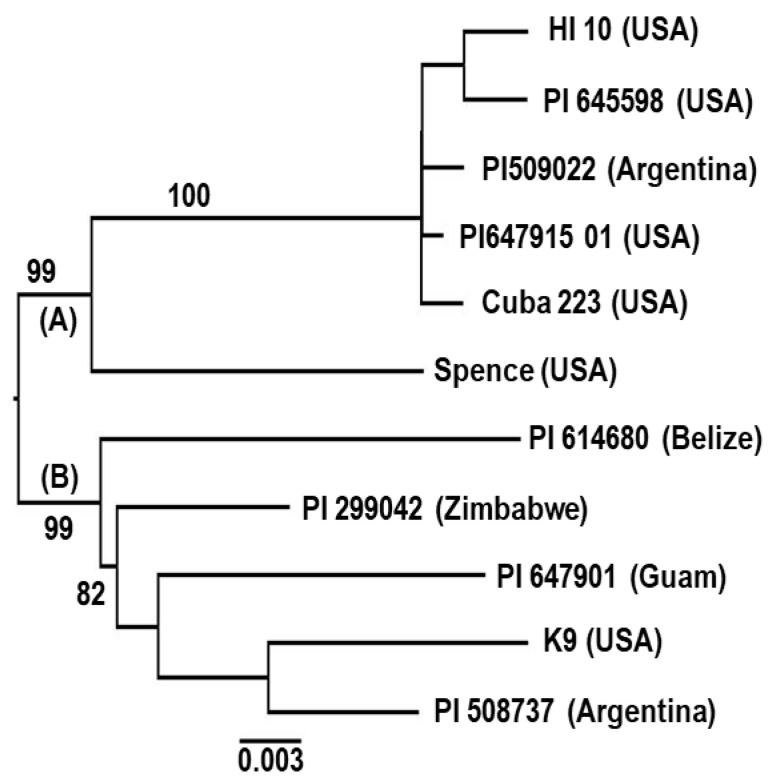
Maximum-likelihood phylogenetic tree based on the full CP nt sequences of PaLV. The branch number indicates bootstrap support in percentage (out of 1000 replicates). The scale bar at the bottom denotes amino acid substitutions per site. The tree is mid-point rooted. The country of origin is shown in parentheses beside the accession numbers. (**A**) and (**B**) denote the two clusters identified in this study: Cluster A, comprising isolates of primarily U.S. origin, and Cluster B, comprising isolates of primarily non-U.S. origin. Although maintained at UGA, the geographical origin is not known for accessions PI647915 01 and K9.

Analysis of eleven CP sequences from PaLV isolates revealed two distinct clusters: Cluster A (primarily U.S. origin) and Cluster B (primarily the rest of the world) (Figure 4). 

F_ST_ value was 0.124, indicating low spatial genetic structure. To ascertain whether the low diversity could be attributed to selection, the dN/dS ratio was estimated, yielding a value of 0.042, suggesting the presence of strong purifying (negative) selection on CP. This finding is further corroborated by the observation of low genetic diversity (0.031 ± 0.004) of CP.

### 2.5. Host Range Studies

All inoculated (virus and mock) plants were examined by RT-PCR after nine indicator plants representing nine plant genera were subjected to mechanical inoculation with leaf extract from PaLV-infected plants. All inoculated plants of the nine assayed genera were monitored over a period of 21 days post-inoculation (dpi) for symptom development. (Supplementary Table S1). PaLV amplicons of the expected size were obtained with RT-PCR in *Zea mays*, *Sorghum* spp., *Setaria italica*, and *Lolium multiflorum*. Interestingly, *T. aestivum* plants maintained at 16 °C were also susceptible to PaLV infection based on RT-PCR. The infection of the aforementioned species indicates they are hosts of PaLV. On the contrary, no amplicons were amplified from the remaining inoculated species: *Hordeum vulgare*, *Avena sativa*, *Dactylis glomerata*, and *Miscanthus sacchariflorus*. None of the experimental hosts showed any symptoms of virus infection. Similarly, no symptoms were observed in infected seashore paspalum germplasm. No symptoms were observed in mock-inoculated plants. In contrast, LoLV infections in *Lolium* plants were reported to be either asymptomatic or to show mild chlorotic streaking on the leaves [25], and in *Nicotiana benthamiana* plants, systemic symptoms such as mosaic and vein netting showed at 15 dpi [26].

### 2.6. Screening of Paspalum Germplasm

Virus-specific primers were designed to target the RNA-dependent RNA polymerase domain in ORF1, amplifying a region of 605 bp. Thirty different *Paspalum* accessions maintained at UGA were analyzed. Two samples of butterfly bush were used as a negative control, and a PaLV-positive plant as a positive control. The RT-PCR results revealed the presence of PaLV infection in 27 out of 30 accessions (Supplementary Table S2), with an incidence of 90% in the tested *Paspalum* accessions. Notably, none of the negative controls showed the presence of PaLV, indicating the specificity of the primer pair.

## 3. Discussion

In this study, we report the molecular and biological characterization of *Paspalum latent virus* (PaLV), a novel virus infecting seashore paspalum and representing a new taxon within the family *Alphaflexiviridae*. The complete genome structure, phylogenetic placement, host range, and population diversity of PaLV were investigated, revealing several unique features that distinguish it from known members of the *Lolavirus* genus. Although PaLV shares moderate sequence identity with *Lolium latent virus* (LoLV), its genome organization diverges significantly. in the replicase, absence of a proteolytic cleavage site within the CP, and the overlapping start-stop codon arrangement between ORF4 and ORF5 suggest distinct evolutionary adaptations. This is a notable divergence from LoLV. Sister members of the PaLV clade, FmLoV1 and Lola maydis, also lack the AlkB domain within the replicase. On the other hand, WaLoV1, which clusters with LoLV contains the AlkB domain. The AlkB domain is speculated to play a crucial role in maintaining the stability of RNA genomes, which are susceptible to methylation due to pesticide applications [25,27]. This speculation led to the hypothesis that the acquisition of the AlkB domain is a relatively recent development in the evolution of viruses within the *Alphaflexiviridae* family [25,27]. Furthermore, the identification of a putative sgRNA2 promoter sequence (GUUAAGUU) 14 nt upstream of the CP gene is consistent with conserved cis-acting elements characterized in related potexviruses such as PVX, BaMV, suggesting that PaLV employs internal initiation mechanisms for CP subgenomic RNA synthesis similar to other *Alphaflexiviridae* members [28,29]. Experimental validation through 5′-RACE would confirm the occurrence and endpoints of CP-specific subgenomic RNA transcripts. PaLV lacks another signature feature of lolaviruses, ORF6, which is also present in WaLoV1. PaLV shows similar protein sizes for the various ORFs to FmLoV1 rather than LoLV and WaLoV1 [22,25]. Overall, the absence and presence of these signature features across both clades further support the divergence across both taxa. As a result, this supports our hypothesis that PaLV represents a new genus, tentatively named ‘*Paspalovirus*’.

Based on the ICTV demarcation criteria in the family *Alphaflexiviridae*, viruses are separated into different genera when their coat protein or polymerase genes share less than about 45% amino acid identity. Viruses are considered distinct species if they share less than 72% nucleotide identity or less than 80% amino acid identity in either their coat protein (CP) or replicase genes. In this study, PaLV replicase showed less than 45% amino acid identity with LoLV, which is below the genus demarcation threshold, and the CP amino acid identity was below 40%. Further, there is incongruency in other features, such as different genome organizations and putative protein sizes, in placing PaLV within the *Lolavirus* or other established genera.

Despite low amino acid identity between PaLV and LoLV CPs, structural modeling revealed some conserved core and C-terminal domains. These domains are analogous to the crystal structures of the CPs of PapMV and *Pepino mosaic virus* from the *Potexvirus* genus [24,30]. The N- and C-terminal extensions play a crucial role in the polymerization of CP [24]. The core and C-terminal regions of the CP play a crucial role in protein-protein and protein-RNA interactions and have been found to show the highest degree of structural conservation in *Alphaflexiviridae* [24]. These regions are critical for virion assembly and RNA binding, suggesting functional conservation. Interestingly, subcellular localization predictions indicated that PaLV CP may localize to cytoplasm or peroxisomes, unlike LoLV and PVX CPs, which preferentially target plastids. This divergence, coupled with a very different N-terminus sequence within PaLV CP compared to LoLV, may influence host interactions and symptom expression. For instance, tentative members FmLoV1 and Lola maydis have several internal smaller ORFs within the CP. Further experimentation beyond the scope of this work is needed to validate whether one or more CP isoforms exist for paspaloviruses.

PaLV was detected in multiple grass species, including *Zea mays*, *Sorghum* spp., *Setaria italica*, and *Lolium multiflorum*, indicating a broad host range. However, no visible symptoms were observed in any infected plants, including paspalum accessions. This latent infection profile complicates field diagnostics and may contribute to unnoticed virus spread through vegetative propagation. In contrast, LoLV infections in *Lolium* plants were reported either to be asymptomatic or showing mild chlorotic streaking on the leaves [25], and in experimental plants, systemic symptoms such as mosaic and vein netting showed at 15 dpi [26]. This indicates that while PaLV shares similarity to LoLV, PaLV does not show severe or adverse effects due to the lack of virulence factors, which can allow the virus to go undetected in some grasses.

Analysis of 11 PaLV isolates revealed low genetic diversity and strong purifying selection on the CP gene. An *F_ST_* of 0.124 indicated a low degree of spatial structure and genetic differences between the population [31,32]. These results are consistent with the expectation of CP sequence conservation given the numerous pivotal functions of CP within *Alphaflexiviridae*, including the activation of RNA translation [33], the facilitation of infection [34], and the encapsidation of viral genome RNA [35]. Our findings are in agreement with studies on viral CP from different viral families that documented strong purifying selection pressure along with low genetic diversity [31,32,36,37]. Altogether, it cannot be ruled out that seashore paspalums may have often been exchanged between countries due to their beneficial use in athletic fields, leading to a low degree of spatial structure in the viral population instead of a very high spatial structure.

Furthermore, because of the small number of accessions analyzed, the fact that all accessions were maintained in a single location for multiple years, and the geographic origins could possibly refer to sites where *Paspalum* plants were maintained before being donated to NPGS [38], whether the two PaLV populations indeed have different geographic origins and distributions will need to be further confirmed. Further, since the plants have been maintained in controlled environments and in pots, it is unlikely that contamination can occur between accessions. Since Paspalum is a vegetatively propagated crop, the transmission of the virus and the lower diversity observed are more likely to be preserved long-term. Similar observations of low-to-high genetic differences were also made in PVX populations, where isolates from different continents were analyzed, where human-mediated movement influenced viral population dynamics [31,32]. Nevertheless, the diversity observed in PaLV should not impact the efficiency of the RT-PCR detection assay developed in this study.

## 4. Materials and Methods

### 4.1. Plant Material and Growth

*Paspalum* plants, obtained initially from the USDA-NPGS collection in Griffin, were maintained in greenhouses at the UGA Griffin and Athens campuses in 4 × 4 pots. The soil was a 1:1 mixture of sand and Miracle-Gro potting mix (The Scotts Miracle-Gro Company, Marysville, OH, USA). Plants were fertilized once every two weeks with Osmocote Plus (ICL Specialty Fertilizers, Summerville, SC, USA).

### 4.2. Total Plant RNA Extraction

Total RNA from 100 mg of *Paspalum* accession PI 509022 leaf tissue was extracted using a RNeasy Plant Mini Kit (Qiagen, Hilden, Germany), and an RNase-Free DNase Set (Qiagen) was used for DNA digestion. The manufacturer’s recommended protocol was used without modifications. For accessions HI10 and Spence, papillae were peeled from the adaxial surface of leaves (the second fully open, developed leaf on the growing stolon segment) from plants grown under freshwater (0 mM NaCl) and salt-stress (200 mM NaCl) irrigation for six weeks. Strings of papillae were collected in 2 mL tubes resting on a liquid nitrogen bath to maintain RNA integrity, and frozen tissues were ground using a TissueLyser II bead mill (Qiagen). The ground tissue was homogenized in 1 mL of TRIzol reagent (Invitrogen, Waltham, MA, USA) per 100 mg of tissue, followed by phase separation after adding 200 μL of chloroform. The RNA-containing aqueous phase was carefully transferred to a 2 mL nuclease-free tube and mixed with an equal volume of 100% ethanol. The resulting RNA-ethanol mixture was loaded onto a Zymo-Spin IC column from the Zymo RNA Clean and Concentrator-5 kit (Zymo Research, Irvine, CA, USA), and RNA clean-up was performed following the manufacturer’s instructions. RNA was eluted in 10 µL nuclease-free water, and RNA concentration was determined using a NanoDrop spectrophotometer (Thermo Fisher Scientific, Waltham, MA, USA). The quality was assessed via electrophoresis on a 1% Tris-Borate-EDTA (TBE) agarose gel.

### 4.3. HTS and Virus Identification

The RNA extracts from leaves of accession PI 509022 at USDA APHIS-PPQ, Beltsville, and from adaxial leaf papillae from accessions HI 10 and Spence at the University of Georgia, Athens, were subjected to HTS at their respective place of extraction. For HTS of RNA from PI 509022, a single-indexed ribosomal RNA-depleted cDNA library was prepared using the TruSeq^®^ Stranded Total RNA Library Prep Plant kit (Illumina, San Diego, CA, USA) and sequenced on an Illumina NextSeq 500 platform, generating 19,815,890 single-end, 75 bp reads. For HTS of RNA from accessions HI 10 and Spence, barcoded stranded mRNA-seq libraries were generated from 500 ng of RNA with the KAPA Stranded mRNA-Seq Kit (Roche, Basel, Switzerland) according to the manufacturer’s instructions, using half-reactions for all steps except the final library amplification. The concentration of the libraries was measured using a Qubit Fluorometer (Invitrogen, Waltham, MA, USA) with the Qubit 1x dsDNA High Sensitivity Assay Kit (Invitrogen). Subsequently, 30 ng of each library was pooled into a larger set of 24 leaf and peel libraries and loaded onto a single flow cell of an Illumina NextSeq 2000 for paired-end (PE) 150 bp sequencing at the Georgia Genomics and Bioinformatics Core (GGBC) at UGA. Reads from HTS were assembled using the de novo assembly tool in PhytoPipe (2023) [39] and compared with viral pathogen databases. Briefly, raw reads were filtered and trimmed using Trimmomatic (v.0.39) (Usadel Lab, RWTH Aachen University, Aachen, Germany) [40]. Then, contigs were assembled using Trinity (v2.8.6) (Broad Institute, Cambridge, MA, USA) [41] and compared to the NCBI Viral Nucleotide Reference Database (Jan 2022) by BLASTn search (NCBI, National Library of Medicine, Bethesda, MD, USA) [42] and to the NCBI Viral Reference Sequence (RefSeq) protein database (v22) (NCBI, Bethesda, MD, USA) [43] by Diamond blastx search (Max Planck Institute for Biophysical Chemistry) [44]. Default parameters were used in all programs except for the e-value threshold, which was set to 1 × 10^−5^.

### 4.4. 5′ RACE

The SMARTer^®^ RACE 5′/3′ Kit (Takara Bio USA, Inc., San Jose, CA, USA) was used to complete the 5′ end of the ssRNA segment with the RACE primer—5′-GGACCACTGTGGCGTAGAGGATGTCGAGGT-3′; the 5′ end of the primer was linked to the adapter sequence provided by the company. All PCR fragments were sequenced by direct Sanger sequencing in both directions, and sequences were aligned to the reference HTS-derived contigs using Geneious (Biomatters, 18 Shortland Street, Auckland, New Zealand) (v11.0.3).

### 4.5. RT-PCR Validation of HTS and Screening of Germplasm

Virus-specific primers were designed to amplify a 605 bp amplicon from the replicase gene. Primer pairs ORF1.FP: 5′-AAACAAGTGGAATTTCACGGG-3′ and ORF1.RP: 5′-CTGCTCTGGTGAGAAGATATCG-3′, were designed using Primer3 software (Whitehead Institute for Biomedical Research, Cambridge, MA, USA). For RT-PCR detection of PaLV, 1 μg of total RNA (extracted as described above) was reverse transcribed using oligo dT primers in a 20 μL reaction with the SuperScript™ III One-Step RT-PCR System with Platinum^®^ Taq DNA Polymerase (Invitrogen), followed by PCR using 1 μL of the resulting cDNA. Cycling parameters followed the manufacturer’s guidelines except for the 62 °C annealing temperature.

### 4.6. Plant Inoculation and Host Screening

After confirmation of PaLV infection in *Paspalum* through RT-PCR, infected plant tissues were freshly ground in 0.01 M potassium phosphate buffer (pH 7) for mechanical inoculation. All plants were sprayed with carborundum, an abrasive, before rub-inoculation. Mechanical inoculation of sap was conducted on nine different plant species: *Hordeum vulgare*, *Triticum aestivum*, *Zea mays*, *Sorghum* spp., *Dactylis glomerata*, *Setaria italica*, *Lolium multiflorum*, *Miscanthus sacchariflorus*, and *Avena sativa*. Three biological replicates were used for each species. All bioassay plants were kept in controlled-temperature greenhouse conditions at 26 °C ± 2 °C with 16 h light and 8 h dark. Two plant species, *H. vulgare* and *T. aestivum*, were assayed in a growth chamber maintained at 16 °C with 16 h light and 8 h dark.

### 4.7. Sequence, Phylogenetic, and Recombination Analysis

The viral sequences were analyzed by BLASTn (https://blast.ncbi.nlm.nih.gov/Blast.cgi) (accessed on 15 November 2024). with default parameters. The putative proteins and potential open reading frames (ORFs) were determined using ORFfinder (https://www.ncbi.nlm.nih.gov/orffinder/) (accessed on 15 November 2024) and subsequent BLASTP annotation (accessed on 15 November 2024) by using the non-redundant protein sequences database. Pairwise comparisons between related viral ORFs were performed with the Sequence Demarcation Tool (SDT) v1.2 to calculate the identity percentage for each ORF (University of Cape Town, Cape Town, South Africa) [45]. Conserved domains within these proteins were identified using the Conserved Domain Database (CDD) [46]. The coat protein (CP) sequences were modeled using AlphaFold2 (Google DeepMind, London, UK) [47]. PaLV CP (CP_PaLV_) was superimposed on the Lolium latent virus (CP_LoLV_) using TM-align [48]. Structural similarity between CP_PaLV_ and CP_LoLV_ was further evaluated based on TM scores, using 0.5 as the cut-off. Superimposed images were exported from TM-align and loaded into Chimera X [49] for further processing.

Evolutionary relationship of PaLV with representative species from the genera, *Lolavirus*, *Potexvirus*, *Mandarivirus*, and *Allexivirus* in the *Alphaflexiviridae* family was included in a maximum-likelihood phylogenetic tree based on amino acid sequences of replicase using the LG with gamma distribution and frequency substitution model previously inferred using jModelTest in MEGA 11 [50]. The phylogenetic analysis was performed with 1000 bootstrap replicates and potato virus T as an outgroup. The resulting phylogenetic tree was visualized and formatted using FigTree v1.4.4 (http://tree.bio.ed.ac.uk/software/figtree/) (accessed on 11 December 2024).

The Recombination Detection Program V.5 (RDP5) program was used to explore the occurrence of recombination events in full-length viral genome sequences. Various methods implemented in RDP5 [51], including RDP, SisterScan, Bootscan, Chimaera, GeneConv, MaxChi, and 3Seq algorithms, were used. Any recombinant events with *p* < 0.001 predicted by four of the seven prediction methods were considered reliable and used in the analysis.

### 4.8. Genetic Diversity

The genetic diversity of the PaLV population was analyzed using 11 PaLV isolates obtained from different *Paspalum* accessions from the UGA collection. Genetic diversity was calculated based on the complete CP gene nucleotide sequences. The primer set designed to amplify the CP region of PaLV was developed according to the consensus sequence of three PaLV isolates obtained through HTS (CP_F: 5′-GATCGGAAGCCTCAGTTGTG-3′; CP_R: 5′-CGGTGGCCAGGGTAGATTAA-3′). The CP nucleotide sequence of 11 PaLV isolates was aligned using the Muscle program implemented in MEGA 11, and the mean genetic diversity for the entire population was calculated. The average number of non-synonymous (d_N_) and synonymous (d_S_) nucleotide substitutions per site and the dN/dS ratio as an estimate of the CP’s selection pressure were computed using the DnaSP v.5 software [52]. The Wright’s fixation index statistic *F_ST_* [53] was calculated using DnaSP v.5. Frequent gene flow is considered to occur when *F_ST_* < 0.33.

## 5. Conclusions

Based on phylogenetic analysis, genomic structure, and comprehensive biological characterization, we propose that PaLV represents a novel species within this new genus, *Paspalovirus*, within the family *Alphaflexiviridae*. While LoLV is the closest recognized virus species to PaLV, it is phylogenetically distinct, and significant differences in genome organization, and the absence of key biological data precludes its classification as a true member of the *Lolavirus*. PaLV was present in most *Paspalum* accessions maintained at UGA. Given that seashore paspalum is typically propagated vegetatively through sod, containerized material, stolons, and rhizomes, the virus can be easily disseminated during plant propagation and potentially readily disseminated by mechanical transmission during mowing [54]. Here, we observed no discernible effect of PaLV infection on *Paspalum*. However, the asymptomatic nature of PaLV infections does not preclude the possibility of its impact on plant growth, breeding, and other plant physiological factors such as salt tolerance, a key horticultural characteristic, and a subject that requires further investigation. Furthermore, it is plausible that further adoption of *Paspalum* in the turf industry can potentially result in the emergence of more virulent PaLV isolates due to environmental changes and viral evolution [55,56,57]. Therefore, PaLV can potentially emerge as a challenge for turfgrass management with the lack of resistance traits in the *Paspalum* germplasm. Further studies should focus on screening *Paspalum* germplasm against PaLV through quantitative and semi-quantitative RT-PCR-based methods, similar to what was observed for resistance to fungal diseases in *Paspalum* [7,58]. Nevertheless, even in the present context, it is essential to implement control methods, such as using virus-free source materials to propagate paspalum.

## Figures and Tables

**Table 1:** Identity (%) in nucleotide and deduced amino acid (aa) sequences between PaLV and other viruses from *Lolavirus, Potexvirus,* and *Mandarivirus* genera.

Virus	Replicase (ORF 1)		TGB1 (ORF2)		TGB2 (ORF3)		TGB3 (ORF4)		CP (ORF5)	
	nt	aa	nt	aa	nt	aa	nt	aa	nt	aa
LoLV	56	44	50	35	57	55	50	31	49	26
PVX	51	43	48	32	47	42	44	25	57	23
BaMV	54	42	48	27	52	44	47	29	54	23
ICRSV	54	39	51	25	52	36	52	40	43	20
CYVCV	53	39	48	27	52	37	54	38	42	21

BaMV: Bamboo mosaic virus (NC_001642); CYVCV: Citrus yellow vein clearing virus (MW429487); ICRSV: Indian citrus ringspot virus (PQ434699); LoLV: Lolium latent virus (EU489641); PVX: Potato Virus X (MT752888).

## Data Availability

The original data presented in the study are openly available in [NCBI] at [www.ncbi.nlm.nih.gov, accessed on 20 June 2026], PV239592 (PaLV isolate Spence), PV239593 (PaLV isolate HI10), PV239594 (PaLV isolate PI509022).

## Supplementary Materials

The following supporting information can be downloaded at: https://www.mdpi.com/article/10.3390/ijms27135760/s1.
