# Supplementary material for: Molecular and Biological Characterization of a Newly Identified Virus Representing a Novel Taxon of Alphaflexiviridae Infecting Different Accessions of Seashore Paspalum, a Turfgrass, Widely Grown in the United States
Source: Int J Mol Sci. 2026 Jun 26;27(13):5760. doi: 10.3390/ijms27135760 (PMC13362480; doi:10.3390/ijms27135760)
Supplement: Supplementary file 1 [file ijms-27-05760-s001.zip › ijms-4342388-supplementary.pdf]

Supplementary Table S1: Bioassay to check for PLV infectivity in different plants species

S.No	Crop	Bioassay Results
1	*Triticum aestivum* (16℃)	+
2	*Hordeum vulgare* (16℃)	-
3	*Triticum aestivum*	-
4	*Hordeum vulgare*	-
5	*Setaria italica*	+
6	*Sorghum spp*'	+
7	*Zea mays*	+
8	*Avena sativa*	-
9	*Lolium multiflorum*	+
10	*Dactylis glomerata*	-
11	*Miscanthus sacchariflorus*	-

Supplementary Table S2: Screening of Paspalum Germplasm to check for PLV infection

Sample	Name	Species [(a)]	From	PLV PCR	Origin [(a)]
1	PI 576140	*P. distichum*	UGA	+	Brazil
2	PI 647922	*P. distichum*	UGA	-	Australia
3	PI 403999	*P. distichum*	UGA	+	Australia
4	Durban	*P. vaginatum*	UGA	+	South Africa
5	PI 614679 01	*P. vaginatum*	UGA	+	Bahamas
6	PI 299042	*P. vaginatum*	UGA	+	Zimbabwe
7	PI 509018-3	*P. vaginatum*	UGA	+	Argentina
8	K9	*P. vaginatum*	UGA	+	NK
9	PI 647920	*P. vaginatum*	UGA	+	Louisiana, USA
10	PI 647912	*P. vaginatum*	UGA	+	Georgia, USA
11	PI 647918	*P. vaginatum*	UGA	+	Florida, USA
12	PI 645598	*P. vaginatum*	UGA	+	Texas, USA
13	PI 647916	*P. vaginatum*	UGA	+	NK
14	Azul	*P. vaginatum*	UGA	-	NK
15	PI 614680	*P. vaginatum*	UGA	+	Belize
16	PI 508737	*P. vaginatum*	UGA	+	Argentina
17	PI647915 01	*P. vaginatum*	UGA	+	Texas, USA
18	PI 647915	*P. vaginatum*	UGA	+	Texas, USA
19	PI 509022	*P. vaginatum*	UGA	+	Argentina
20	Cuba 223	*P. vaginatum*	UGA	+	Hawaii, USA
21	Q36315	*P. vaginatum*	UGA	+	Israel
22	PI 647908 01	*P. vaginatum*	UGA	+	Georgia, USA
23	PI 377709 02	*P. vaginatum*	UGA	+	South Africa
24	Kim 1	*P. vaginatum*	UGA	+	Georgia, USA
25	PI 647901	*P. vaginatum*	UGA	+	Guam
26	Hyb 5	*P. vaginatum*	UGA	+	NA
27	Sea Dwarf	*P. vaginatum*	UGA	-	Florida, USA
28	KC 9	*P. vaginatum*	UGA	+	Texas, USA
29	Spence	*P. distichum* [(b)]	UGA	+	Louisiana, USA
30	HI 10	*P. vaginatum* [(b)]	UGA	+	Hawaii, USA

(a) – The information on *Paspalum* species except for Spence and HI 10 and their origin were obtained from Eudy et al., 2017

(b) – The information on *Paspalum* species were obtained from Spiekerman and Devos, 2020
